# Identifying molecular targets of Aspiletrein-derived steroidal saponins in lung cancer using network pharmacology and molecular docking-based assessments

**DOI:** 10.1038/s41598-023-28821-8

**Published:** 2023-01-27

**Authors:** Iksen Iksen, Wasita Witayateeraporn, Tanakrit Wirojwongchai, Chutipa Suraphan, Natapol Pornputtapong, Natsaranyatron Singharajkomron, Hien Minh Nguyen, Varisa Pongrakhananon

**Affiliations:** 1 Department of Pharmacology and Physiology, Faculty of Pharmaceutical Sciences, Chulalongkorn University, 254 Phayathai, Wangmai, Pathumwan, Bangkok, 10330 Thailand; 2 Department of Pharmacy, Sekolah Tinggi Ilmu Kesehatan Senior Medan, Medan, Indonesia; 3 Department of Biochemistry and Microbiology, Faculty of Pharmaceutical Sciences, and Center of Excellence in Systems Biology, Faculty of Medicine, Chulalongkorn University, Bangkok, Thailand; 4 Faculty of Pharmacy, Ton Duc Thang University, Ho Chi Minh City, Vietnam; 5 Preclinical Toxicity and Efficacy Assessment of Medicines and Chemicals Research Unit, Chulalongkorn University, Bangkok, Thailand

**Keywords:** Cancer, Computational biology and bioinformatics, Drug discovery

## Abstract

Lung cancer is one of the leading cancers and causes of cancer-related deaths worldwide. Due to its high prevalence and mortality rate, its clinical management remains a significant challenge. Previously, the in vitro anticancer activity of Aspiletrein A, a steroid and a saponin from *Aspidistra letreae*, against non-small cell lung cancer (NSCLC) cells was reported. However, the anticancer molecular mechanism of other Aspiletreins from *A. letreae* remains unknown. Using in silico network pharmacology approaches, the targets of Aspiletreins were predicted using the Swiss Target Prediction database. In addition, key mediators in NSCLC were obtained from the Genetic databases. The compound-target interacting networks were constructed using the STRING database and Cytoscape, uncovering potential targets, including STAT3, VEGFA, HSP90AA1, FGF2, and IL2. Gene Ontology and Kyoto Encyclopedia of Genes and Genomes pathway analysis demonstrated that several pathways were highly relevant to cancer pathogenesis. Additionally, molecular docking and molecular dynamic analyses revealed the interaction between key identified targets and Aspiletreins, including hydrogen bonding and Van der Waals interaction. This study provides potential targets of Aspiletreins in NSCLC, and its approach of integrating network pharmacology, bioinformatics, and molecular docking is a powerful tool for investigating the mechanism of new drug targets on a specific disease.

## Introduction

Lung cancer remains an aggressive malignancy with high mortality and incidence rate because of its aggressive characteristics^1^. Among the common subtypes of lung cancer, non-small cell lung cancer (NSCLC) is the primary subtype, accounting for 85% of the cases^2^. The standard therapy for NSCLC includes surgery, radiation, chemotherapy, targeted therapy, and immunotherapy^3^. In the past decade, despite advances in therapeutic approaches, the number of deaths has gradually increased due to NSCLC’s ability to metastasize, acquisition of chemotherapeutic resistance, and high recurrence rate; in addition, drug efficacy is limited by significant side effects^4,5^. Particularly, a five-year survival rate of only 7% was reported for patients with lung cancer at the metastatic stage^1^. Therefore, continuous drug discovery for lung cancer is necessary for improving the clinical outcome.

Plants are a versatile source of biologically active compounds. For example, the plants of the *Aspidistra* genus, discovered in Vietnam^6^, contain many active components, such as saponin, coumarin, and isoflavones^7,8^, with various demonstrated pharmacological activities, including antibacterial, antifungal, antitumor, and antiviral^7–10^. A previous study reported that Aspiletreins, mainly three steroidal saponins, exhibited cytotoxicity in breast, cervical, hepatocellular, gastric, and lung cancer cells^8,11^. These three steroidal saponins contain different sugar moiety side chains located at C3, suggesting distinct potencies (Table 1). The mechanistic investigation demonstrated that Aspiletrein A (AA) could suppress lung cancer metastasis by inhibiting protein kinase A (Akt) signaling^11^. However, the molecular targets of Aspiletreins B and C (AB and AC, respectively) have not been elucidated.

**Table 1:** Compound information.

No.	Parameters	Aspiletrein A	Aspiletrein B	Aspiletrein C
1	MW	857.044	1019.185	1149.328
2	Log P	0.9331	− 1.2427	− 1.3633
3	H-bond acceptor	16	21	24
4	H-bond donors	8	11	12
5	Rotatable bond	7	10	11
6	% Intestinal absorption	63.78	39.771	25.623
7	BBB (log BB)	− 0.28	− 2.125	− 2.533
8	Ames toxicity	No	No	No
9	Hepatotoxicity	No	No	No
10	Max. tolerated dose (mg/kg/day)	0.007	0.122	0.348
11	Drug likeness	N/A	N/A	N/A
12	Oral bioavailability	N/A	N/A	N/A

On the other hand, network pharmacology-based strategies, integrating complex systems of biology and the network analysis of multiple drug targets, have been established as a new paradigm for drug discovery^12^. This type of approach is a powerful tool for predicting the molecular targets of new chemical entities as well as disease pathways. In addition, molecular docking analysis reveals the potential intermolecular interactions between a target and compounds^13^. Furthermore, pharmacokinetics and pharmacodynamics were analyzed according to chemical properties and structures using the algorithm-based method^14^. These comprehensive approaches can help reduce the time and cost of preclinical experiments. In this study, we explored the molecular mechanism of Aspiletreins against NSCLC using network pharmacology and computation-based approaches.

## Results

### Identification of drug targets in non-small cell lung cancer

**Figure 1:**
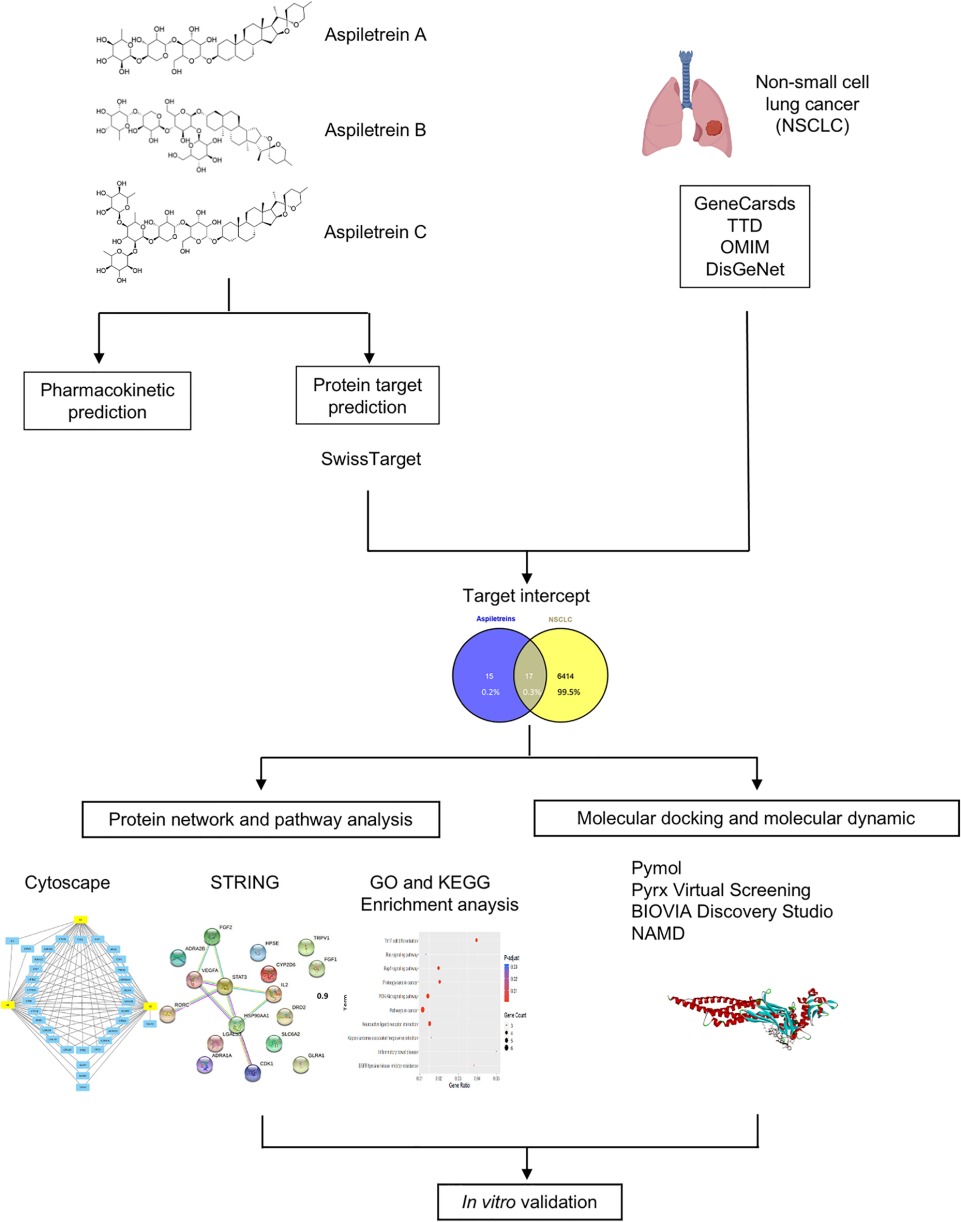
The framework of this study.

**Figure 2:**
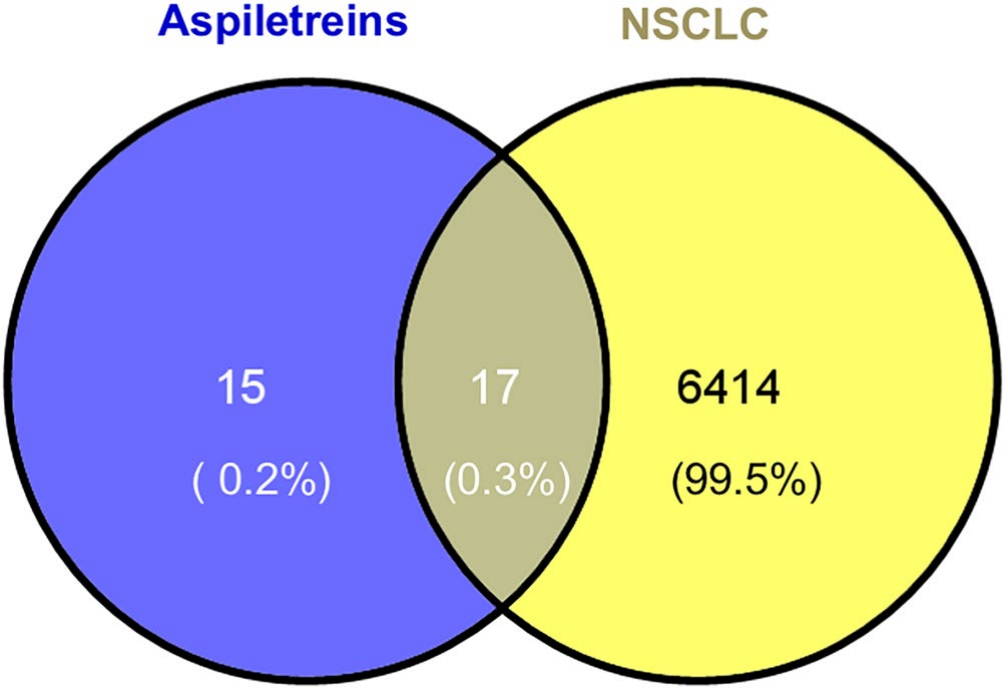
Venna diagram representing the overlapping of NSCLC targets (yellow) and compound targets (blue).

**Figure 3:**
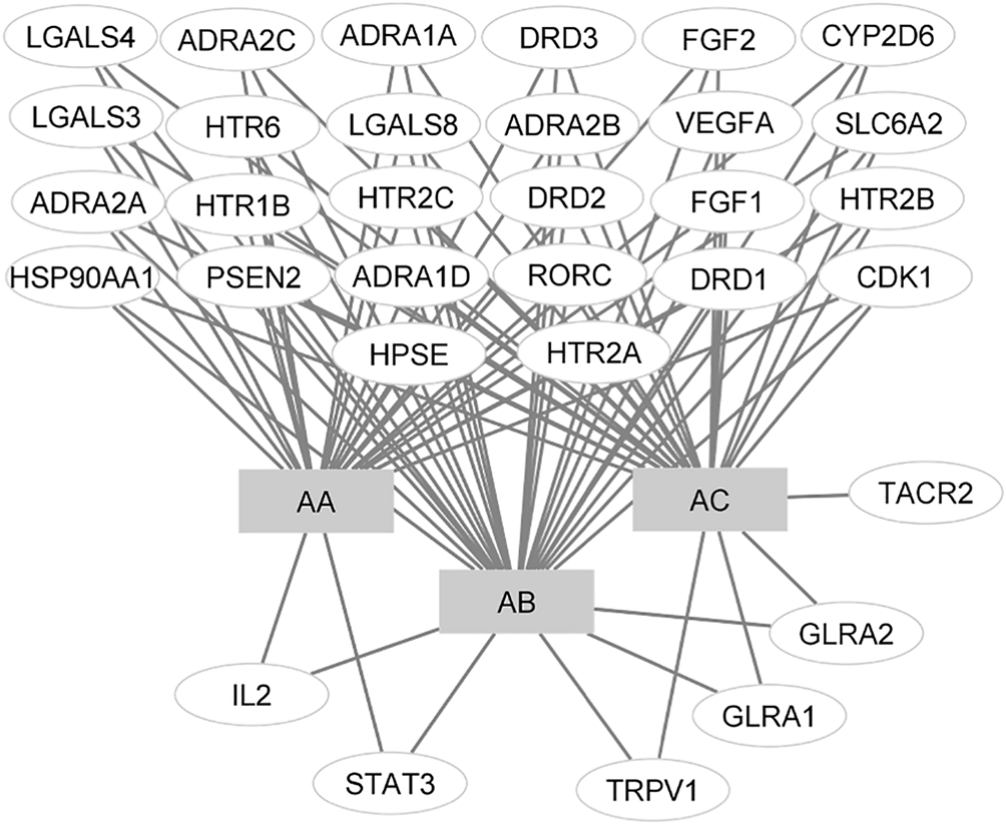
Compound-target-NSCLC network constructed using Cytoscape v_3.9.0. The grey rectangles represent the compounds (AA, AB, and AC), and the white ovals represent the hub protein-target interaction. *AA* Aspiletrein A, *AB* Aspiletrein B, *AC* Aspiletrein C.

The experiment procedures are presented in Fig. 1. Targets in NSCLC were searched in the GeneCards Human database, DisGeNET, OMIM, and TTD using “non-small cell lung cancer” as a keyword. After removing duplication among these databases, a total of 6431 NSCLC targets were found based on the relevance scores (Fig. 2). The targets of each Aspiletrein were fetched from the Swiss Target Prediction. The predicted targets identified included 28 for AA, 32 for AB, and 30 for AC (Table 2). The 17 intersecting targets of Aspiletreins and NSCLC were in a Venn diagram (Fig. 2) and listed (Table S1). Furthermore, the compound-target network plot was constructed using Cytoscape 3.9.0 (Fig. 3). Among the targets, STAT3 and IL2 for AA and AB, GLRAs and TRPV1 for AB and AC, whereas TACR2 was a target only for AC.

**Table 2:** Lists of compound targets obtained from the Swiss Target Prediction.

No.	Aspiletrein A (AA)	Aspiletrein B (AB)	Aspiletrein C (AC)
1	VEGFA	VEGFA	HSP90AA1
2	FGF1	FGF1	PSEN2
3	FGF2	FGF2	CDK1
4	HPSE	HPSE	HPSE
5	CDK1	HSP90AA1	VEGFA
6	LGALS4	PSEN2	FGF1
7	LGALS8	CDK1	FGF2
8	HSP90AA1	LGALS4	HTR2B
9	HTR2B	LGALS8	ADRA2A
10	ADRA2A	HTR2B	ADRA2C
11	ADRA2C	ADRA2A	ADRA2B
12	ADRA2B	ADRA2C	DRD1
13	DRD1	ADRA2B	HTR2C
14	DRD2	DRD1	CYP2D6
15	ADRA1D	DRD2	HTR6
16	HTR2A	ADRA1D	HTR1B
17	HTR2C	HTR2A	RORC
18	DRD3	HTR2C	LGALS4
19	CYP2D6	DRD3	LGALS3
20	HTR6	CYP2D6	LGALS8
21	HTR1B	HTR6	DRD2
22	PSEN2	ADRA1A	DRD3
23	LGALS3	HTR1B	ADRA1A
24	RORC	LGALS3	ADRA1D
25	IL2	RORC	TRPV1
26	ADRA1A	TRPV1	HTR2A
27	STAT3	SLC6A2	TACR2
28	SLC6A2	STAT3	SLC6A2
29		GLRA1	GLRA1
30		GLRA2	GLRA2
31		IL2	
32		VEGFA

### Analysis of target protein–protein interaction (PPI) network

**Figure 4:**
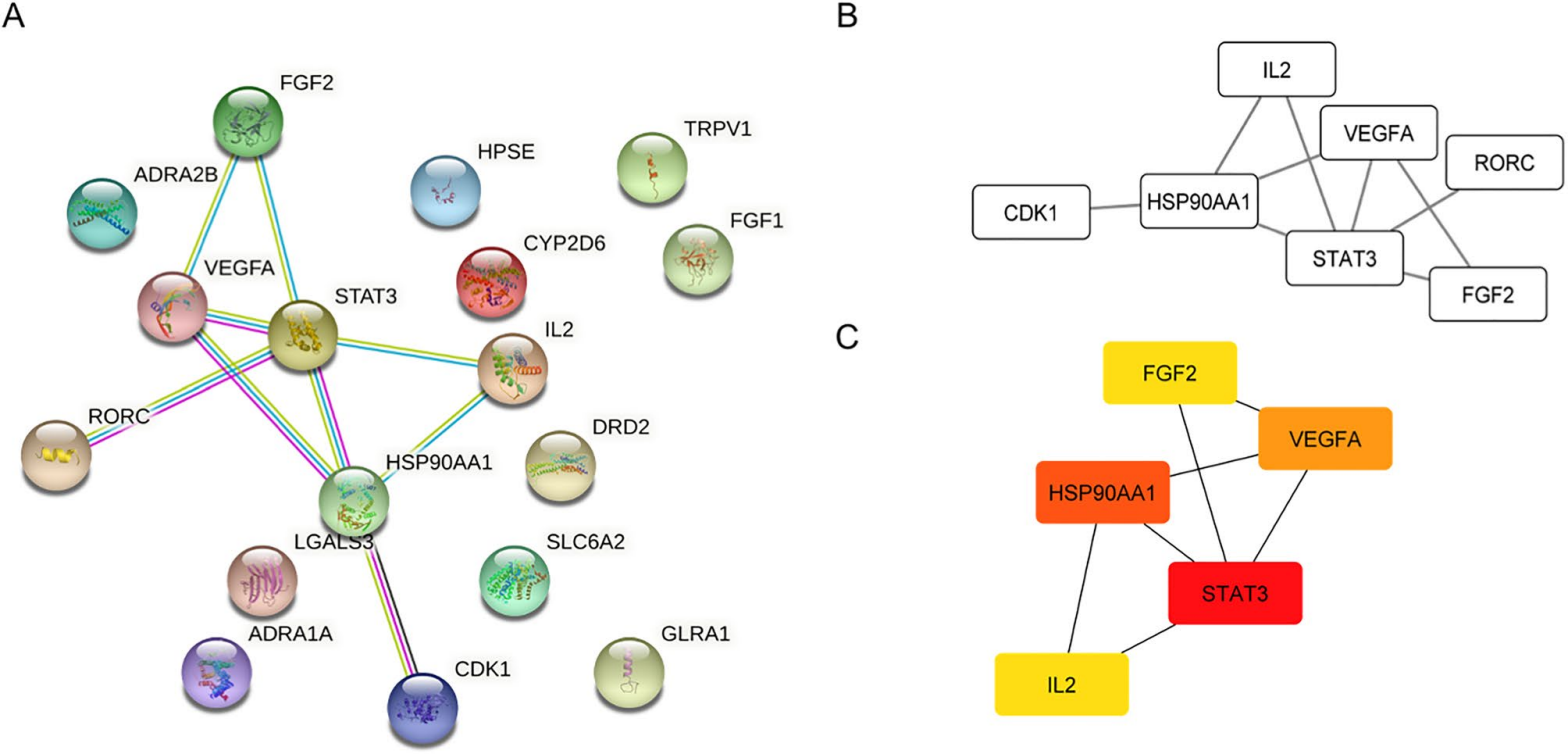
Protein–protein interaction (PPI) analysis. (**A**) Protein–protein interaction network of Aspiletreins and NSCLC targets obtained from STRING v_11.5 database. (**B**) The PPI network was constructed using the plug-in targets from the STRING database and imported into Cytoscape. (**C**) The top 5 targets in the PPI network as ranked using the cytoHubba plug in network analyzer. The higher degree value is represented by colors ranging from red to yellow.

The 17 intersected targets, including VEGFA, FGF1, FGF2, HPSE, CDK1, HSP90AA1, ADRA2B, DRD2, CYP2D6, LGALS3, RORC, IL2, ADRA1A, STAT3, TRPV1, SLC6A2, TRPV1, and GLRA1, were imported to the STRING database, and the potential relationships among them were investigated. The PPI network with a confidence score of 0.9 was constructed (Fig. 4A). The STRING database analysis revealed that the average node degree, defined as the average number of interactions of a protein in a network, was 1.06 and the local clustering coefficient, defined as the wellness of the connected nodes in a network, was 0.312. The interactions between the targets comprised 17 nodes and 9 edges, with each edge representing the association between nodes. Cytoscape analysis showed that there was one main cluster associated in the PPI network (Fig. 4B). According to the scores of degree, closeness centrality, betweenness centrality, and clustering centrality, signal transducer and activator of transcription 3 (STAT3), heat shock protein HSP 90-alpha (HSP90AA1), vascular endothelial growth factor A (VEGFA), fibroblast growth factor-2 (FGF2), and interleukin-2 (IL-2) were identified as the top 5 intersecting targets of Aspiletreins and NSCLC interaction (Fig. 4C and Table S2).

### Gene Ontology (GO) and Kyoto Encyclopedia of Genes and Genomes (KEGG) pathway analysis

**Figure 5:**
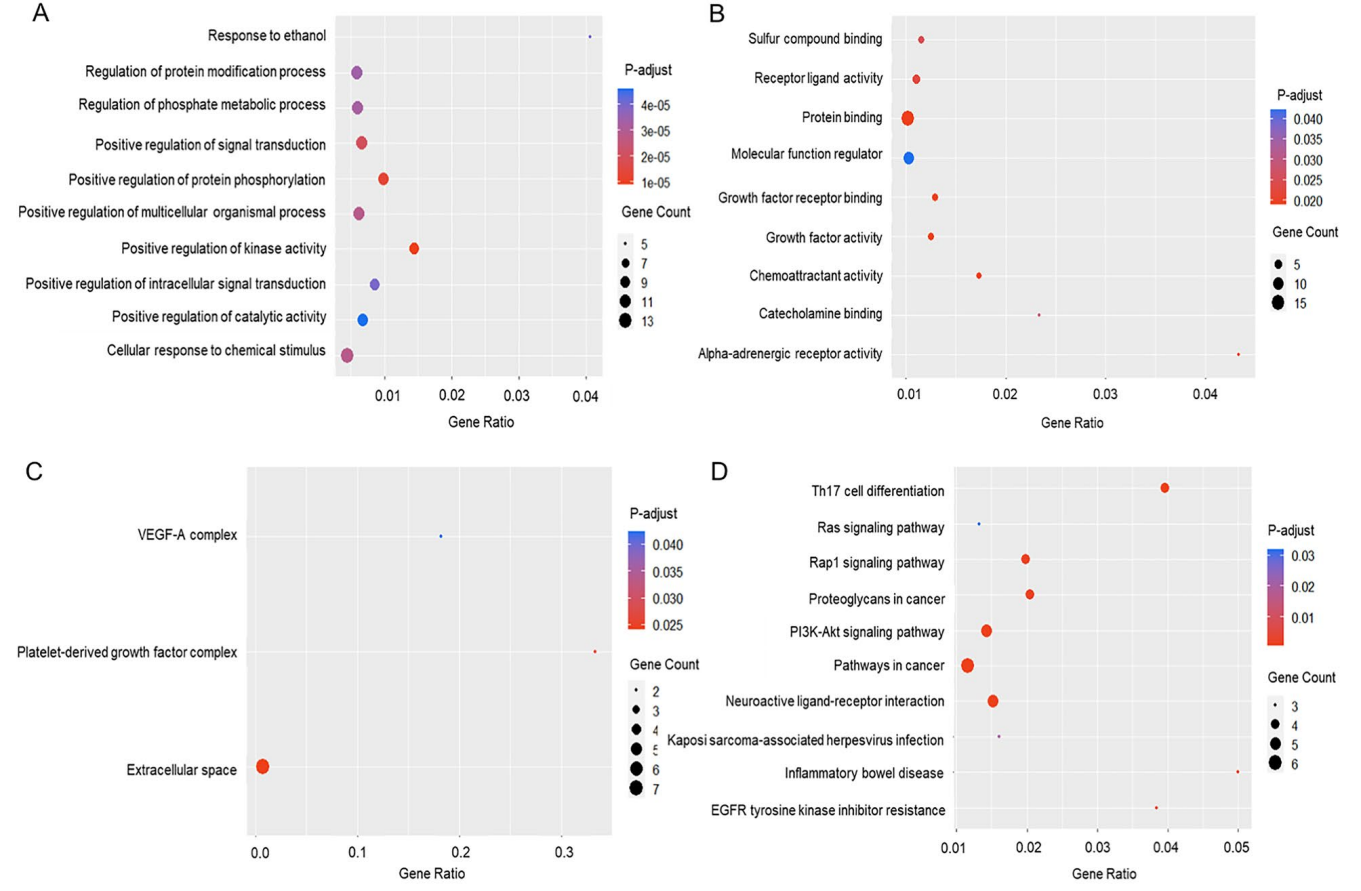
Gene Ontology (GO) and Kyoto Encyclopedia of Genes and Genomes (KEGG) pathway enrichment analysis. The biological processes (**A**), molecular functions (**B**), subcellular localization (**C**), and KEGG terms (**D**) distributed in the ordinate and the degree of enrichment were analyzed. The size of the dots represents the gene count. Adjusted *P*-value indicates the importance of enrichment in which the blue-to-red represents the high-to-low value of the enrichment.

GO enrichment analysis was conducted to analyze the impact of the targets in NSCLC. The classification of GO was set for three criteria, including biological processes (Fig. 5A), GO molecular functions (Fig. 5B), and GO subcellular localizations (Fig. 5C). First, the data with the candidate target and KEGG pathway analysis were interpreted using the R software with a ggplot2 plug-in, uncovering 245 biological processes, 9 molecular functions, and 3 subcellular localizations. Then, the top 10 biological processes of Aspiletreins were identified by sorting them by the degree of significance, including response to ethanol, regulation of protein modification process, regulation of phosphate metabolic process, positive regulation of signal transduction, positive regulation of protein phosphorylation, positive regulation of multicellular organismal process, positive regulation of kinase activity, positive regulation of intracellular signal transduction, positive regulation of catalytic activity, and cellular response to chemical stimulus (Fig. 5A). Molecular functions were mainly enriched in growth factor activity, chemoattractant activity, growth factor receptor binding, protein binding, receptor-ligand activity, molecular function regulator, sulfur compound binding, catecholamine binding, and alpha-adrenergic receptor activity (Fig. 5B). Finally, subcellular localization includes extracellular space, platelet-derived growth factor complex, and VEGF-A complex (Fig. 5C). Furthermore, the KEGG pathway suggested that the predicted targets were components of the pathways involved in oncogenesis, particularly in the PI3K-Akt, Rap1, Ras, and MAPK signaling pathways, and resistance to EGFR tyrosine kinase inhibitors (Fig. 5D, Fig. S1).

### Compounds-target interaction analysis by molecular docking and molecular dynamic simulation

**Figure 6:**
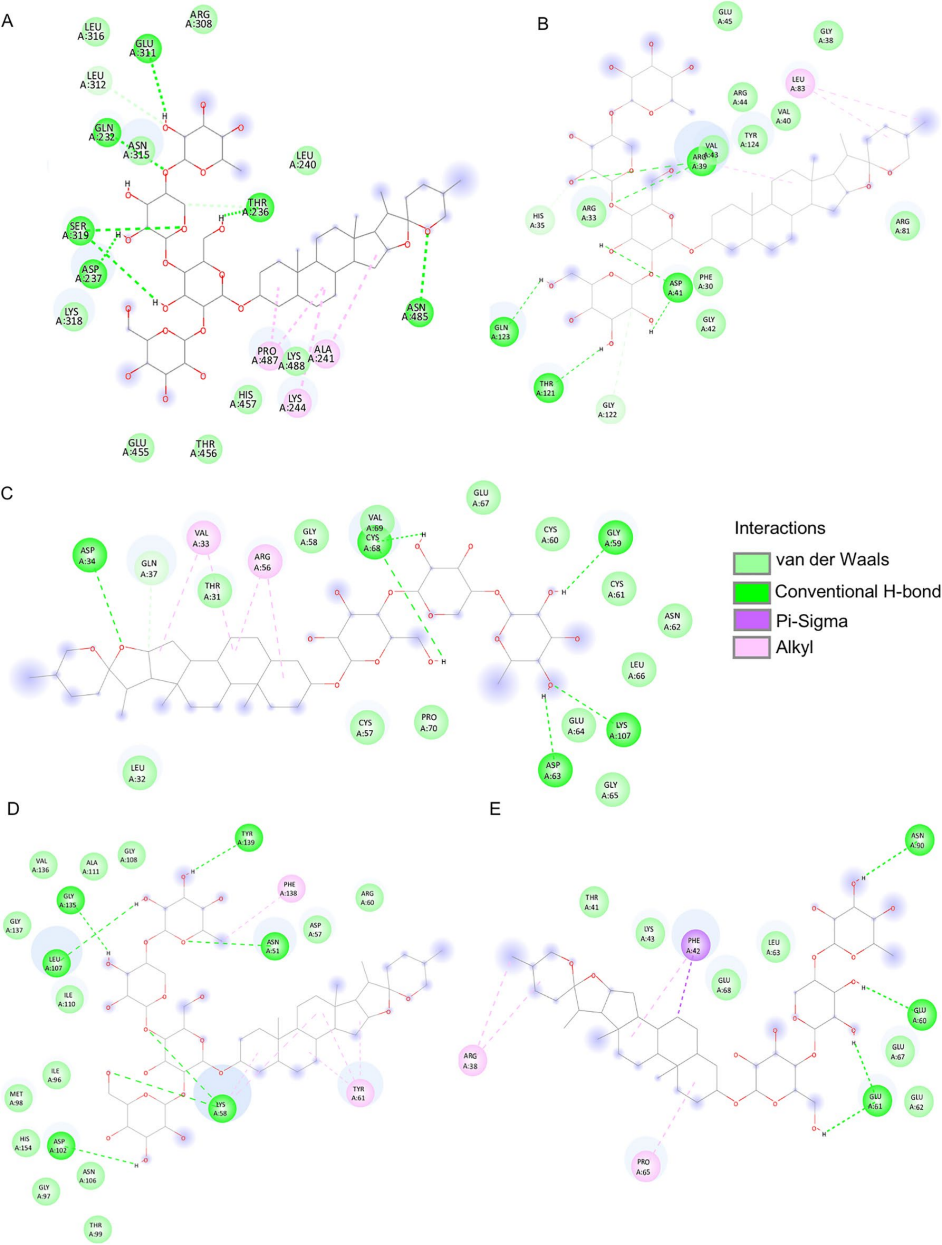
Molecular docking between Aspiletreins and the top 5 targets that have the highest affinity. (**A**) The interaction between AA and STAT3 (PDB: 6NJS). (**B**) The interaction between AA and VEGFA (PDB: 4KZN). (**C**) The interaction between AB and HSP90AA1 (PDB: 4BQG). (**D**) The interaction between AB and FGF2 (PDB: 2FGF). (**E**) The interaction between AA and IL2 (PDB: 1M48). Hydrogen bonds were displayed in green, and Van der Waals interactions were displayed in light green. Alkyls were displayed in pink, and π–sigma was displayed in purple. *AA* Aspiletrein A, *AB* Aspiletrein B.

Among the 17 main targets of Aspiletreins in NSCLC, the top 5 potential targets, including STAT3, VEGFA, HSP90AA1, FGF2, and IL-2, were investigated for possible interaction with Aspiletreins. The Aspiletreins were molecularly docked using the PyRx Virtual Screening Tool, and their interactions with the highest affinity in each target were presented (Fig. 6, Fig. S2). The major binding interactions included hydrogen bonding and Van der Waals interactions. The binding energy score during docking indicates the affinity of a component for the target protein^15^. Here, all of the binding energy scores analyzed were less than 0, suggesting high-affinity interactions among the targets and the Aspiletreins (AA, AB, and AC) (Table. 3).

**Table 3:** Binding energy among the compounds and the top 5 potential targets.

Compound	Binding energy (kcal/mol)				
	STAT3	VEGFA	HSP90AA1	FGF2	IL-2
AA	− 9.1	− 8	− 8	− 6.9	− 7.7
AB	− 9.3	− 7.1	− 9	− 7.2	− 7.5
AC	–	− 7.7	− 7.3	− 6.9	–

**Figure 7:**
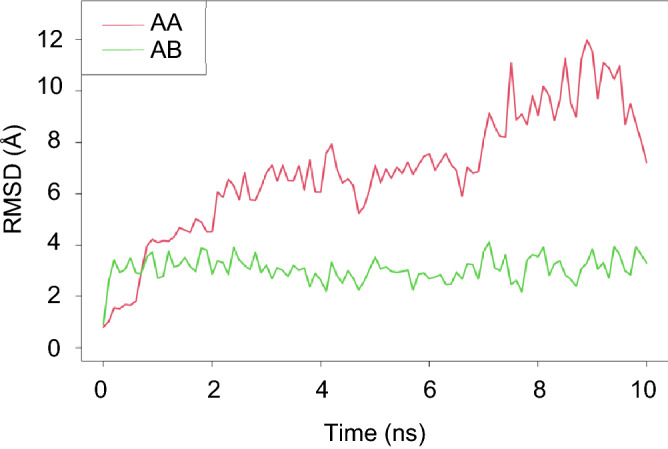
Molecular dynamic interaction between AA and AB with STAT3 during 0–10 ns. The RMSD for ligand movement plot for interaction of AA (red) or AB (green) with STAT3 complex.

Since our finding demonstrated that STAT3 exhibited the most potential target of compounds in lung cancer cells, molecular dynamic simulation between STAT3 and AA or AB was then performed. A RMSD plot generated by R-Studio was used to evaluate the residual deviations in the complexes. The data shows that both AA and AB have comparable consistent interaction with STAT3 over the time after their binding (Fig. 7, Videos S1 and S2). However, the ligand movement in the binding pocket side of STAT3 showed that AB was more stable due to its longer time in the aspect of equilibrium, which average RMSD of AB was 3.07 ± 0.49 Å, whereas that of AA was 6.87 ± 2.4 Å. Apart from RMSD plot, other factors such as the amount of hydrogen, hydrophobic, and Van der Waals interaction between ligand and protein are important to determine the efficacy of ligand–protein interaction^16^. In this case, AB has more significant hydrogen interaction (Table S3) compared to AA, in which hydrogen bonds play a major role in the stabilization of ligand–protein complexes^17^. These results suggest the stable behaviors of the complexes formed between AA or AB with STAT3.

### In vitro cytotoxicity and target validation

Cytotoxicity of the tested compounds was performed in NSCLC-H460 cells by MTT assay. The results demonstrated that IC_50_ of AA, AB, and AC in H460 cells were 13.25 ± 0.77, 6.82 ± 0.87, and 15.76 ± 0.17 μM respectively (Fig. S3A), indicating that AB has the most potent compound. Next, an in vitro experiment for validating the molecular target of the compounds was examined. Since STAT3 plays an important role in cancer survival and apoptosis, and our finding demonstrated that STAT3 was the most relevant core target (Fig. 4, Table S2), the effect of AB on STAT3 activity was then investigated. The data demonstrated that phosphorylated STAT3 (pSTAT, an active form) was significantly decreased in response to AB, whereas its total form was unchanged (Fig. S3B), confirming the molecular mechanism identified. This suggests that network pharmacology in combination with molecular docking approaches is a beneficial platform for the identification of new drugs.

## Discussion

Despite the advances in cancer treatment, such as chemotherapy, radiotherapy, targeted therapy, and immunotherapy, lung cancer continues to be one of the main causes of cancer-related deaths worldwide and a major global health problem due to its low five-year overall survival rate and poor prognosis^1^. Natural compounds are a promising source of drug candidates for lung cancer treatment^18^; therefore, they are investigated for their potential in improving clinical overcome. Recently, our group discovered Aspiletreins A, B, and C, promising natural compounds from *Aspidistra letreae*. In addition, these steroidal saponins reportedly exhibited cytotoxicity in various cancer cell lines^8,11^. In this study, we have identified the molecular mechanism between the Aspiletreins and their predicted targets using the network pharmacology approach in combination with in silico molecular docking.

Network pharmacology-based assessments provide in-depth analyses on how various network factors interact with potential drug candidates^19–21^. This approach has emerged as a powerful tool for drug target identification based on a multidisciplinary concept integrating biology systems and polypharmacological models^22^. Aside from discovering new drugs and their targets, this tool enables the exploration of prospective target areas and the repurposing of existing drugs for diverse diseases^23^.

In this study, the potential targets of the compounds that overlapped with NSCLC targets were identified, including STAT3, VEGFA, HSP90AA1, FGF2, and IL2. These targets are highly relevant in the progression and metastasis in lung cancer^24–29^. STAT3, one of the main members of the STAT family, is responsible for the transcription of several genes related to oncogenesis, such as those encoding the proteins of the CDK family and pro-survival Bcl-2 family, VEGF, and epithelial-to-mesenchymal transition-related transcription factors SNAIL and SLUG^25,30^. Even though STAT3 expression was not significantly altered in tumor (Fig. S4A), the STAT3 mutation was strongly correlated to the overall survival rate of lung cancer patients (Fig. S4B), and hyperphosphorylation of STAT3 was extensively found in lung adenocarcinoma (Fig. S4G), suggesting that suppression of its phosphorylation provides promising therapeutic approach^31,32^. Meanwhile, VEGFA, a growth factor, functions particularly through VEGFR-1 and VEGFR-2 on endothelial cells. It has various modes of action, including forming new blood vessels and vascular networks, increasing vascular permeability, stimulating endothelial cell proliferation and migration, and preventing endothelial cell apoptosis^26,33^. On the other hand, *HSP90AA1* is a gene that regulates the expression of heat shock protein 90α (HSP90α) in response to intracellular stress^27^. In addition to its intracellular roles, HSP90α acts as an inflammation-stimulated, secreted extracellular factor that promotes malignant cell motility and metastasis by activating NF-kB and STAT3 transcription programs^27^. In the case of growth factor FGF2, it possesses broad mitogenic functions under normal conditions, especially in embryonic development and tissue repairment. In contrast, in cancer, its gene becomes overexpressed, inducing uncontrolled proliferation and the metastasis of several malignant tumors^29,34^. In addition, IL2, a multifunctional glycoprotein of the interleukin family, is a growth factor involved in the immune system, promoting the growth of natural killer cells, B-cells, and T-cells^35,36^. IL2 also aids the immune system by improving the ability of specific white blood cells to identify and destroy cancer cells^37^. In lung cancer tissues, low expression of IL2 was associated with poor prognosis (Fig. S4C). Even though some of them, their expressions were not directly relevant to the overall survival rate of NSCLC patients (Fig. S4), they participate in cancer signaling as downstream molecules or ligands of growth factor receptors whose upstream regulators or receptors were aberrant expressed or overactivated in cancers^38–41^. Furthermore, targeting these core targets was widely reported as potential therapeutic strategy for cancer^42–44^. Taken together with previous study, the mechanisms of these molecular targets may inform on the mechanisms of Aspiletreins against cancer^8,11^.

These promising targets were investigated for their interactions with Aspiletreins using molecular docking analysis. Consistent with our hypothesis, the Aspiletreins bound to the targets with high affinity and likely inhibited the tumor-promoting functions of STAT3, VEGFA, HSP90AA1, and FGF2 or enhanced tumor-suppressing activity of IL2, or both. In addition, the cytotoxicity of these compounds was shown, which AB posed the most potent toxic to lung cancer cells, and STAT3 was validated as a potential target of this compound. Although advances in the field of biological systems and network pharmacology can aid rational drug design and development as well as target identification^45,46^, the information of new compounds including Aspiletreins are not completely available in several databases, the interactions between drugs and their targets need to be further elucidated, and pharmacokinetic profile and the safety and efficacy of the drugs need to be verified. This work, at least, full filled the scientific information of this compound suggesting their potential for anti-cancer drug research and development for clinical application.

## Conclusion

Using network pharmacology and molecular docking-molecular dynamic approaches, this study systematically analyzes the pharmacological mechanism of Aspiletreins in the treatment of NSCLC. Aspiletreins suppress NSCLC by targeting various proteins, including STAT3, VEGFA, HSP90AA1, FGF2, and IL-2. The network pharmacology approach has significant advantages for uncovering the mechanism of Aspiletreins, and narrowing down the number of targets, thus minimizing the time and cost of preclinical investigations. Further in vitro and in vivo experiments will be conducted to verify the mode of action of Aspiletreins against NSCLC.

## Methods

### Compound database

The compounds information of Aspiletrein A (AA), Aspiletrein B (AB), and Aspiletrein C (AC) were identified using the J-GLOBAL Database (https://jglobal.jst.go.jp/en), while the log P information (Table 1) was obtained from the pkCSM tools (http://biosig.unimelb.edu.au/pkcsm/), an online pharmacokinetic prediction model.

### Compounds-target identification

The canonical SMILE of the compounds was uploaded into the Swiss Target Prediction database (http://www.swisstargetprediction.ch/)^47^. The compound-target network was visualized using Cytoscape (3.9.0)^48^.

### Non-small cell lung cancer target identification

The NSCLC-related targets were retrieved by searching for “non-small cell lung cancer” in the GeneCards database (https://www.genecards.org/), DisGeNET (https://www.disgenet.org/), OMIM (https://www.omim.org/), and TTD (http://db.idrblab.net/ttd/). The targets of NSCLC and compounds (AA, AB, and AC) were integrated using VENNY (https://bioinfogp.cnb.csic.es/tools/venny_old/index.html) and the intersected targets were presented in a Venn diagram.

### Construction of protein-PPI network

A PPI network was constructed by uploading the genes to the STRING v_11.0 database (https://string-db.org/)^49^. The settings for building the PPI network were established in accordance with the “*Homo sapiens*” model, and the confidence of the interaction between the targets was set at 0.9^50^. The network nodes represented proteins, and the edges reflected the protein–protein interactions. For the core target protein identification, the Cytoscape data was sorted by using these parameters, including degree, closeness centrality, betweenness centrality, and clustering coefficient.

### Gene Ontology (GO) and Kyoto Encyclopedia of Genes and Genomes (KEGG) analysis

Gene Ontology (GO)^51^ and Kyoto Encyclopedia of Genes (KEGG)^52^ were analyzed by plotting the data analysis obtained from STRING 11.5 database as a scatterplot to display the relationship between two continuous variables of gene count and adjusted *P*-value. The R software with ggplot2^53^ was applied to construct a bubble scatterplot of the GO (molecular function, biological function, and subcellular localization) and KEGG biological pathway. The target count and significance value (*P* < 0.05) were mapped to size and color, respectively.

### Molecular docking

The possible interaction between compounds AA, AB, and AC and the targets, including STAT3 (PDB ID: 6NJS), VEGFA (PDB ID: 4KZN), HSP90AA1 (PDB ID: 4BQG), FGF2 (PDB ID: 2FGF), and IL-2 (PDB ID: 1M48), were modeled. Each protein was retrieved from RCSB Protein Data Bank (https://www.rcsb.org/pages/policies). The interaction between each compound with its target was predicted using PyRx Virtual Screening Tools (Version 0.8)^54^. All the interactions among the target proteins and compounds were constructed using PyMOL 2.5 in the PDBQT format file^55^. Lastly, the 2-dimensional visualization of the interaction between compounds and target proteins was investigated using Discovery Studio Visualizer 2021.

### Molecular dynamic simulation

SwissParam web service^56^ was used to create topology and parameter in CHARMM36 force field (version Jul 21)^57^ for AA and AB ligands. The topology and parameter of STAT3 complexes were patched and generated using pfsgen (version 2.0). The complexes were solvated by TIP3 water^58^ in 15 Å for each direction and ionized using VMD’s solvate and autoIonize plugins (version 1.9.2)^59^. The simulations were performed using NAMD (version 2.14)^60^. The simulation system was minimized for 1000 steps the simulated for 50,000 steps, 2 fs for each step using particle mesh Ewald, Langevin dynamic simulation under 300 K and 1 atm. The rmsd of molecular dynamic simulation were calculated using VMD’s NAMD Energy and RMSD Trajectory Tool plugins (version 1.9.2)^59^.

## Supplementary Information

Supplementary Information.

Supplementary Video S1.

Supplementary Video S2.

## Data Availability

The datasets used and/or analyzed during the current study are included in this published article and its supplementary information files.

## References

[1] Siegel RL, Miller KD, Fuchs HE, Jemal A (2021). Cancer statistics, 2021. CA Cancer J. Clin..

[2] Rodriguez-Canales J, Parra-Cuentas E, Wistuba II (2016). Diagnosis and molecular classification of lung cancer. Cancer Treat. Res..

[3] Duma N, Santana-Davila R, Molina JR (2019). Non-small cell lung cancer: Epidemiology, screening, diagnosis, and treatment. Mayo Clin. Proc..

[4] Herbst RS, Morgensztern D, Boshoff C (2018). The biology and management of non-small cell lung cancer. Nature.

[5] Rotow J, Bivona TG (2017). Understanding and targeting resistance mechanisms in NSCLC. Nat. Rev. Cancer.

[6] Lý NS, Tillich HJ (2016). *Aspidistra averyanovii* and *A. parviflora* (Asparagaceae), two new species Central Vietnam. Phytotaxa.

[7] Liang XX, Kong LX, Fei WB, He M (2018). Chemical constituents and antibacterial activities of *Aspidistra typica*. Chin. J. Nat. Med..

[8] Ho DV (2020). Three new steroidal saponins from *Aspidistra letreae* plants and their cytotoxic activities. J. Nat. Med..

[9] Koketsu M, Kim M, Yamamoto T (1996). Antifungal activity against food-borne fungi of *Aspidistra elatior* blume. J. Agric. Food Chem..

[10] Xu XC (2015). Antiviral and antitumor activities of the lectin extracted from *Aspidistra elatior*. Z. Naturforsch. C..

[11] Nguyen HM (2021). Antitumor activities of Aspiletrein A, a steroidal saponin from *Aspidistra*
*letreae*, on non-small cell lung cancer cells. BMC Complement. Med. Ther..

[12] Hopkins AL (2008). Network pharmacology: The next paradigm in drug discovery. Nat. Chem. Biol..

[13] Pinzi L, Rastelli G (2019). Molecular docking: Shifting paradigms in drug discovery. Int. J. Mol. Sci..

[14] Daina A, Michielin O, Zoete V (2017). SwissADME: A free web tool to evaluate pharmacokinetics, drug-likeness and medicinal chemistry friendliness of small molecules. Sci. Rep..

[15] Kato K, Nakayoshi T, Fukuyoshi S, Kurimoto E, Oda A (2017). Validation of molecular dynamics simulations for prediction of three-dimensional structures of small proteins. Molecules.

[16] Hevener KE (2009). Validation of molecular docking programs for virtual screening against dihydropteroate synthase. J. Chem. Inf. Model..

[17] Chen D (2016). Regulation of protein-ligand binding affinity by hydrogen bond pairing. Sci. Adv..

[18] Iksen, Pothongsrisit S, Pongrakhananon V (2021). Targeting the PI3K/AKT/mTOR signaling pathway in lung cancer: An update regarding potential drugs and natural products. Molecules.

[19] Hopkins AL (2007). Network pharmacology. Nat. Biotechnol..

[20] Yildirim MA, Il Goh K, Cusick ME, Barabási AL, Vidal M (2007). Drug-target network. Nat. Biotechnol..

[21] Lai X (2020). Editorial: Network pharmacology and traditional medicine. Front. Pharmacol..

[22] Zhang R, Zhu X, Bai H, Ning K (2019). Network pharmacology databases for traditional Chinese medicine: Review and assessment. Front. Pharmacol..

[23] Ye H, Wei J, Tang K, Feuers R, Hong H (2016). Drug repositioning through network pharmacology. Curr. Top. Med. Chem..

[24] Huynh J, Chand A, Gough D, Ernst M (2019). Therapeutically exploiting STAT3 activity in cancer—using tissue repair as a road map. Nat. Rev. Cancer.

[25] Dutta P, Sabri N, Li J, Li WX (2015). Role of STAT3 in lung cancer. JAK-STAT.

[26] Frezzetti D (2016). Vascular endothelial growth factor A regulates the secretion of different angiogenic factors in lung cancer cells. J. Cell. Physiol..

[27] Zuehlke AD, Beebe K, Neckers L, Prince T (2015). Regulation and function of the human HSP90AA1 gene. Gene.

[28] Donnem T, Al-Shibli K, Al-Saad S, Busund LT, Bremnes RM (2009). Prognostic impact of fibroblast growth factor 2 in non-small cell lung cancer: Coexpression with VEGFR-3 and PDGF-B predicts poor survival. J. Thorac. Oncol..

[29] Li L (2018). FGF2 and FGFR2 in patients with idiopathic pulmonary fibrosis and lung cancer. Oncol. Lett..

[30] Huang S (2007). Regulation of metastases by signal transducer and activator of transcription 3 signaling pathway: Clinical implications. Clin. Cancer Res..

[31] Li R (2013). Niclosamide overcomes acquired resistance to erlotinib through suppression of STAT3 in non-small cell lung cancer. Mol. Cancer Ther..

[32] Lee JH, Kim C, Sethi G, Ahn KS (2015). Brassinin inhibits STAT3 signaling pathway through modulation of PIAS-3 and SOCS-3 expression and sensitizes human lung cancer xenograft in nude mice to paclitaxel. Oncotarget.

[33] Álvarez-Aznar A, Muhl L, Gaengel K (2017). VEGF receptor tyrosine kinases: Key regulators of vascular function. Curr. Top. Dev. Biol..

[34] Akl MR (2016). Molecular and clinical significance of fibroblast growth factor 2 (FGF2 /bFGF) in malignancies of solid and hematological cancers for personalized therapies. Oncotarget.

[35] Roy U (2019). Structure and function of an inflammatory cytokine, interleukin-2, analyzed using the bioinformatic approach. Protein J..

[36] Mortara L (2018). Anti-cancer therapies employing IL-2 cytokine tumor targeting: Contribution of innate, adaptive and immunosuppressive cells in the anti-tumor efficacy. Front. Immunol..

[37] Jiang T, Zhou C, Ren S (2016). Role of IL-2 in cancer immunotherapy. Oncoimmunology.

[38] Yang P, Xiong J, Zuo L, Liu K, Zhang H (2018). miR-140-5p regulates cell migration and invasion of non-small cell lung cancer cells through targeting VEGFA. Mol. Med. Rep..

[39] Liang L (2019). Autophagy inhibition potentiates the anti-angiogenic property of multikinase inhibitor anlotinib through JAK2/STAT3/VEGFA signaling in non-small cell lung cancer cells. J. Exp. Clin. Cancer Res..

[40] Semrad TJ, Mack PC (2012). Fibroblast growth factor signaling in non-small-cell lung cancer. Clin. Lung Cancer.

[41] Liu K (2018). TRPM7 overexpression enhances the cancer stem cell-like and metastatic phenotypes of lung cancer through modulation of the Hsp90α/uPA/MMP2 signaling pathway. BMC Cancer.

[42] Zhan S, Wang C, Yin F (2018). MicroRNA-29c inhibits proliferation and promotes apoptosis in non-small cell lung cancer cells by targeting VEGFA. Mol. Med. Rep..

[43] He L, Meng Y, Zhang Z, Liu Y, Wang X (2018). Downregulation of basic fibroblast growth factor increases cisplatin sensitivity in A549 non-small cell lung cancer cells. J. Cancer Res. Ther..

[44] Kim WY, Oh SH, Woo JK, Hong WK, Lee HY (2009). Targeting heat shock protein 90 overrides the resistance of lung cancer cells by blocking radiation-induced stabilization of hypoxia-inducible factor-1alpha. Cancer Res..

[45] Atanasov AG (2021). Natural products in drug discovery: Advances and opportunities. Nat. Rev. Drug Discov..

[46] Harvey AL, Edrada-Ebel R, Quinn RJ (2015). The re-emergence of natural products for drug discovery in the genomics era. Nat. Rev. Drug Discov..

[47] Daina A, Olivier M, Vincent Z (2019). SwissTargetPrediction: Updated data and new features for efficient prediction of protein targets of small molecules. Nucleic Acids Res..

[48] Shannon P (2003). Cytoscape: A software environment for integrated models of biomolecular interaction networks. Genome Res..

[49] Szklarczyk D (2019). STRING v11: Protein–protein association networks with increased coverage, supporting functional discovery in genome-wide experimental datasets. Nucleic Acids Res..

[50] von Mering C (2005). STRING: Known and predicted protein-protein associations, integrated and transferred across organisms. Nucleic Acids Res..

[51] Ashburner M (2000). Gene ontology: Tool for the unification of biology. The Gene Ontology Consortium. Nat. Genet..

[52] Kanehisa M, Goto S (2000). KEGG: Kyoto encyclopedia of genes and genomes. Nucleic Acids Res..

[53] Ito K, Murphy D (2013). Application of ggplot2 to pharmacometric graphics. CPT Pharmacom. Syst. Pharmacol..

[54] Dallakyan S, Olson AJ (2015). Small-molecule library screening by docking with PyRx. Methods Mol. Biol..

[55] Seeliger D, De Groot BL (2010). Ligand docking and binding site analysis with PyMOL and Autodock/Vina. J. Comput. Aided Mol. Des..

[56] Zoete V, Cuendet MA, Grosdidier A, Michielin O (2011). SwissParam: A fast force field generation tool for small organic molecules. J. Comput. Chem..

[57] Best RB (2012). Optimization of the additive CHARMM all-atom protein force field targeting improved sampling of the backbone φ, ψ and side-chain χ(1) and χ(2) dihedral angles. J. Chem. Theory Comput..

[58] Mark P, Nilsson L (2001). Structure and dynamics of the TIP3P, SPC, and SPC/E water models at 298 K. J. Phys. Chem. A.

[59] Humphrey W, Dalke A, Schulten K (1996). VMD: Visual molecular dynamics. J. Mol. Graph..

[60] Phillips JC (2020). Scalable molecular dynamics on CPU and GPU architectures with NAMD. J. Chem. Phys..

