# Supplementary material for: Identifying molecular targets of Aspiletrein-derived steroidal saponins in lung cancer using network pharmacology and molecular docking-based assessments
Source: Sci Rep. 2023 Jan 27;13:1545. doi: 10.1038/s41598-023-28821-8 (PMC9883450; doi:10.1038/s41598-023-28821-8)
Supplement: Supplementary file 1 — Supplementary Information. [file 41598_2023_28821_MOESM1_ESM.docx]

***Supplementary information***

**Identifying molecular targets of Aspiletrein*-*derived steroidal saponins in lung cancer using network pharmacology and molecular docking-based assessments**

Iksen Iksen^1^, Wasita Witayateeraporn^1^, Tanakrit Wirojwongchai^1^, Chutipa Suraphan^1^, Natapol Pornputtapong^2^, Natsaranyatron Singharajkomron^1^, Hien Minh Nguyen^3^, Varisa Pongrakhananon^1,4*^

^1^ Department of Pharmacology and Physiology, Faculty of Pharmaceutical Sciences, Chulalongkorn University, Bangkok, Thailand

^2^ Department of Biochemistry and Microbiology, Faculty of Pharmaceutical Sciences, and Center of Excellence in Systems Biology, Faculty of Medicine, Chulalongkorn University, Bangkok, Thailand

^3^ Faculty of Pharmacy, Ton Duc Thang University, Ho Chi Minh City, Vietnam

^4^ Preclinical Toxicity and Efficacy Assessment of Medicines and Chemicals Research Unit, Chulalongkorn University, Bangkok, Thailand

***Corresponding author**

Varisa Pongrakhananon, Department of Pharmacology and Physiology,

Faculty of Pharmaceutical Sciences, Chulalongkorn University, 254 Phayathai, Wangmai, Pathumwan, Bangkok, Thailand 10330

Tel: +662-218-8325; Fax: +662-218-8340

Email: [Varisa.p@pharm.chula.ac.th](mailto:Varisa.p@pharm.chula.ac.th)

***Supplementary materials and methods***

**Overall survival analysis of targets**

The mRNA expression, genetic mutation, and clinical data regarding the lung adenocarcinoma of The Cancer Genome Atlas (TCGA) database were obtained from cBioPortal (<https://www.cbioportal.org/>). The Kaplan-Meier plots, Log-Rank *p­*-values, and hazard ratios were analyzed to compare the overall survival rate between patients with high and low mRNA expression (divided by median), or patients with wild type and mutant of the target genes by using GraphPad Prism 9. The phosphoprotein expression of STAT3 compared between normal and tumor lung samples of the Clinical Proteomic Tumor Analysis Consortium (CPTAC) database was analyzed by UALCAN (<http://ualcan.path.uab.edu/>).

**Cell culture**

Non-small cell lung cancer H460 cells were purchased from American Type Culture Collection (ATCC). Cells were culture in RPMI-1640 supplemented with 10% fetal bovine serum albumin, 2 mM L-glutamine, and 100 U/mL penicillin-streptomycin and maintained in 5% CO_2_ at 37°C.

**Cytotoxicity assay**

Cytotoxicity was examined by using the MTT assay. H460 cells were seeded at 8 × 10^3^ cells/well into 96-well plates. After attachment, cells were treated with various concentrations of AA, AB, or AC for 24 h, and 10 μL of MTT (5 mg/mL) was then added to each well. After incubation for 4 h, 100 μL of DMSO was added to dissolve the formazan, and the optical density was measured using a microplate reader at 570 nm.

**Western blot analysis**

H460 cells were plated at 10^6^ cells onto a 60-mm dish for 24 h and treated with 25 and 50 μM AB for 24 h. Cells were lysed with TMEN lysis buffer (20 mM Tris-HCl pH 7.5, 1 mM MgCl_2_, 150 mM NaCl, 20 mM NaF, 1% octylphenoxypolyethoxyethanol, 0.1 mM phenylmethylsulfonyl fluoride, 0.5% sodium deoxycholate, and cOmplete^TM^ Protease inhibitor Cocktail (Sigma-Aldrich, MO, US) on ice for 30 min. The supernatant was collected by centrifugation at 12,000 × *g* at 4 °C for 15 min. Protein content was quantified using a BSA protein assay kit (Thermo Fisher Scientific Inc, MA, USA). An equal amount of total protein was separated using SDS-polyacrylamide gel electrophoresis and transferred to polyvinyl difluoride membranes. The membranes were then blocked in 5% skim milk in TBS with 0.075% tween for 1 h and incubated with anti-STAT3, anti-phosphorylated STAT3 or anti-GAPDH at 4°C overnight. After washing with TBS-T, and membranes were incubated with a secondary antibody for 2 h at room temperature. Protein expression was visualized using a chemiluminescent HRP substrate (Millipore, MA, USA), and protein bands were quantified using ImageJ software (NIH).

***Supplementary Figure***


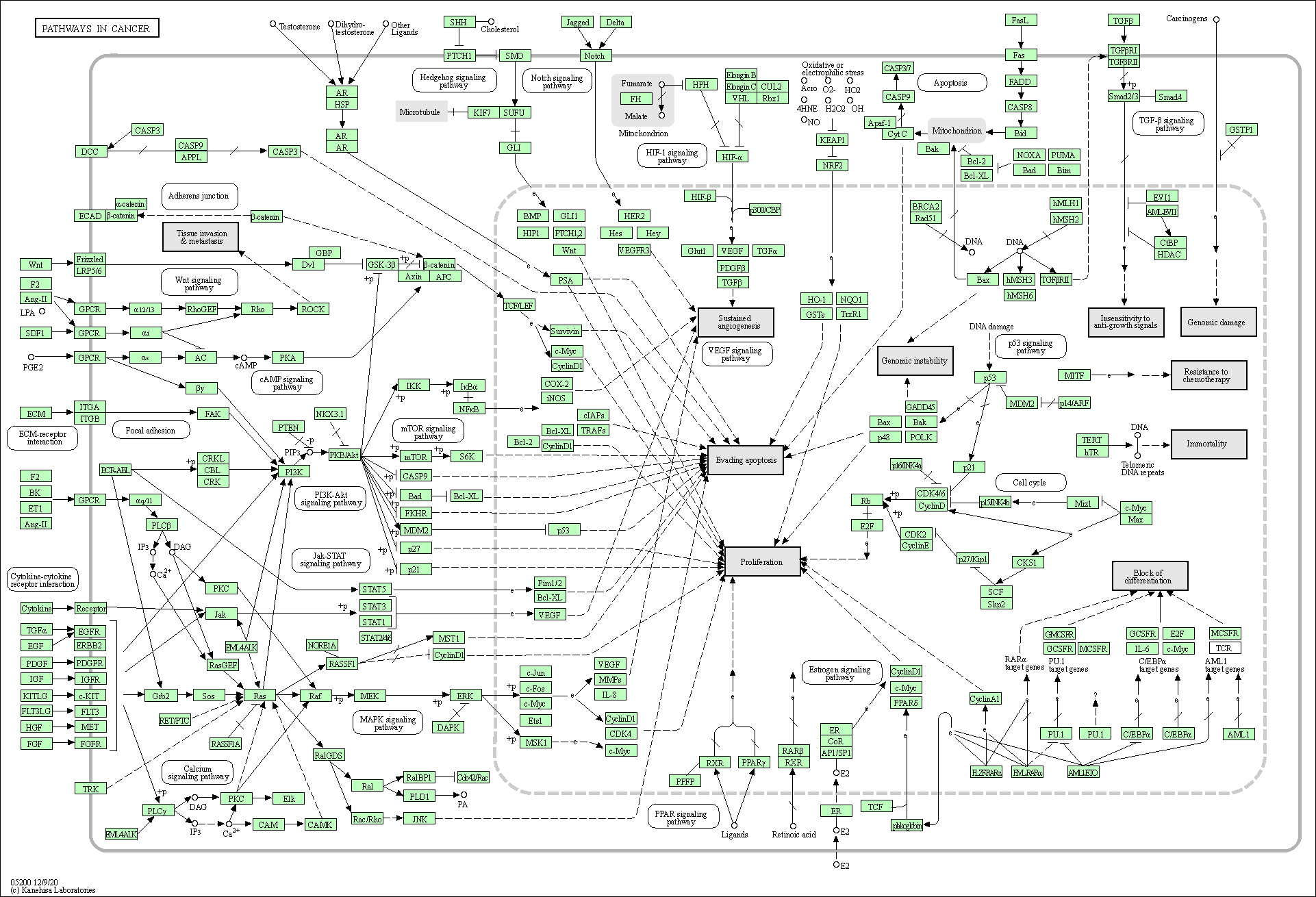


**Figure S1** Relevant targets of Aspiletreins (red rectangle) and signaling pathways in cancer.

**
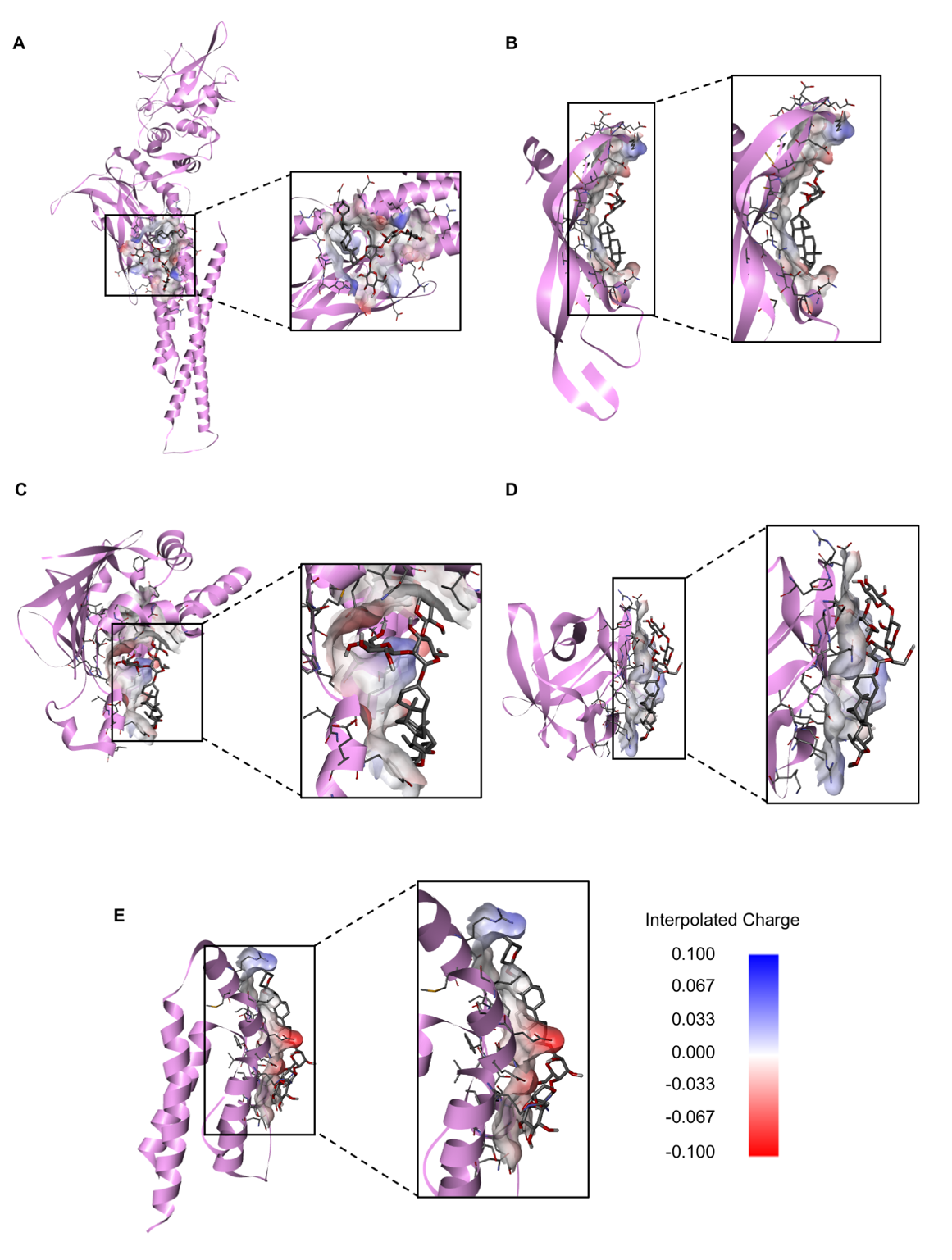
**

**Figure S2** The 3D molecular docking between Aspiletreins and the top 5 targets that have the highest affinity. (A) The interaction between AA and STAT3. (B) The interaction between AA and VEGFA. (C) The interaction between AB and HSP90AA1. (D) The interaction between AB and FGF2. (E) The interaction between AA and IL2. AA, Aspiletrein A; AB, Aspiletrein B.

**
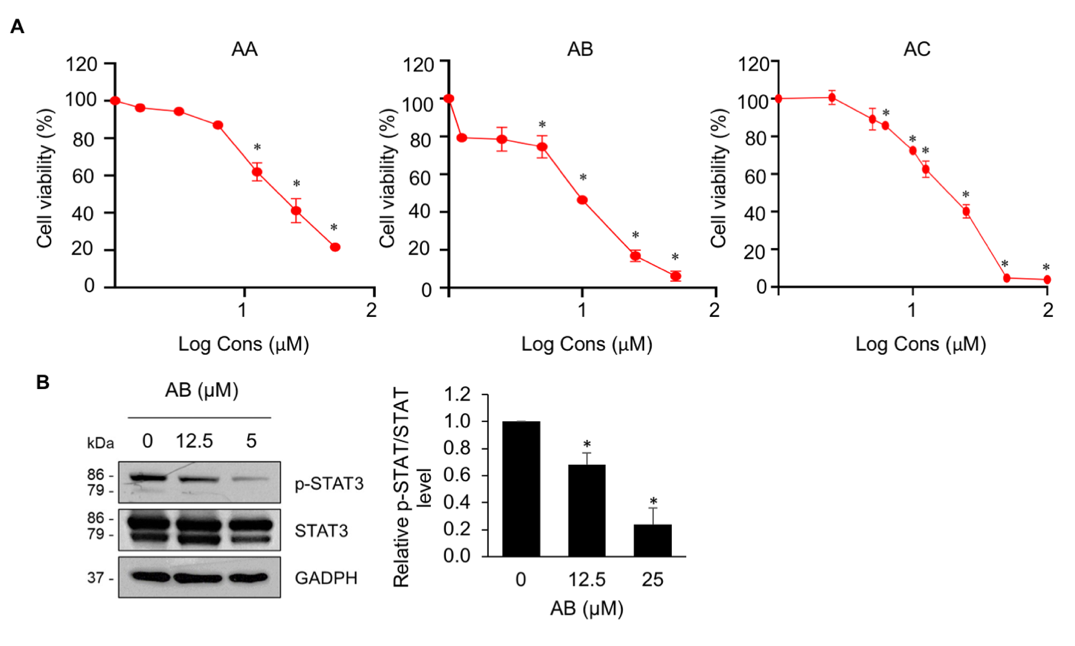
**

**Figure S3** *In vitro* cytotoxicity and target validation. (A) H460 cells were treated with 0-100 μM of AA, AB or AC for 24 h. Cell viability was examined by MTT assay. (B) STAT3 and phosphorylated STAT3 (p-STAT3) expressions were investigated by Western blot analysis. Blots were reprobed with anti-GADPH to confirm equal loading. Protein level was quantified and presented as relative value to the control. All data are mean ± SD (n = 3). **p* < 0.05 vs control cells.

**
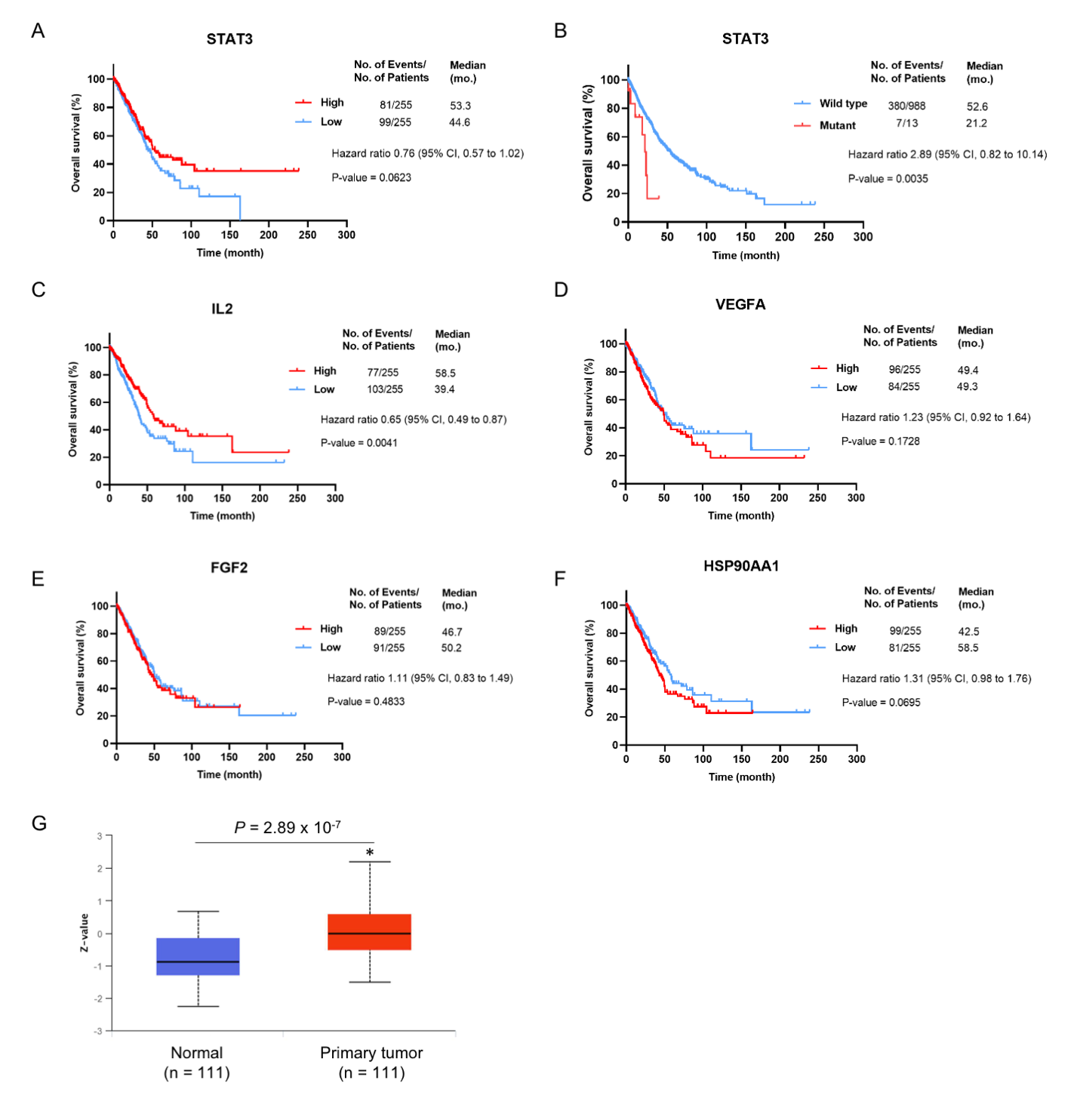
**

**Figure S4** Overall survival analysis of (A) STAT3 expression, (B) STAT3 mutation, (C) IL2 expression, (D) VEGFA expression, (E) FGF2 expression, and (F) HSP90AA1 expression in lung adenocarcinoma tissues. (G) The expression level of phosphorylated STAT3 in normal lung and primary lung adenocarcinoma tissues.

**
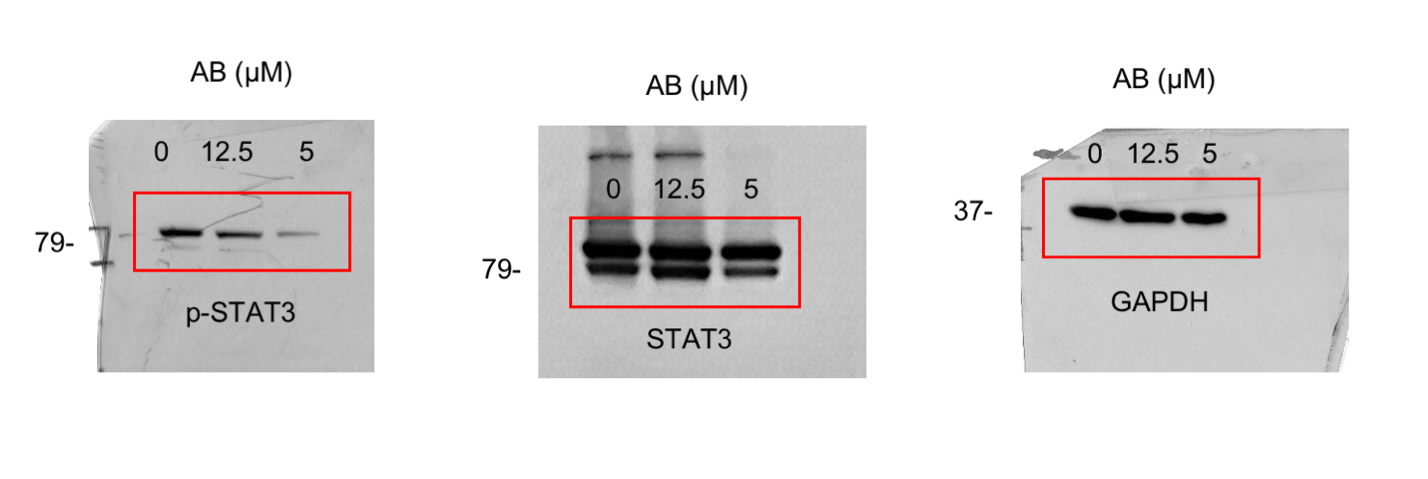
**

**Figure S4** Original blots for Fig S 3B.

***Supplementary Table***

**Table S1. List of intercept targets between Aspiletreins (AA, AB, and AC) and non-small cell lung cancer cell (NSCLC) targets**

| **No** | **Intercept targets** |
| --- | --- |
| 1 | VEGFA |
| 2 | FGF1 |
| 3 | FGF2 |
| 4 | HPSE |
| 5 | CDK1 |
| 6 | HSP90AA1 |
| 7 | ADRA2B |
| 8 | DRD2 |
| 9 | CYP2D6 |
| 10 | LGALS3 |
| 11 | RORC |
| 12 | IL2 |
| 13 | ADRA1A |
| 14 | STAT3 |
| 15 | SLC6A2 |
| 16 | TRPV1 |
| 17 | GLRA1 |

**Table S2. Important nodes in network analyzer**

| **Name** | **Degree** | **Betweenness Centrality** | **Closeness Centrality** | **Clustering Coefficient** |
| --- | --- | --- | --- | --- |
| STAT3 | 5 | 0.5 | 0.857142857 | 0.3 |
| HSP90AA1 | 4 | 0.366666667 | 0.75 | 0.333333333 |
| VEGFA | 3 | 0.066666667 | 0.666666667 | 0.666666667 |
| FGF2 | 2 | 0 | 0.545454545 | 1 |
| IL2 | 2 | 0 | 0.6 | 1 |
| CDK1 | 1 | 0 | 0.461538462 | 0 |
| RORC | 1 | 0 | 0.5 | 0 |

**Table S3. Amino Acids interaction between AA and AB with STAT3**

| **Protein** | **Compound** | **Binding interaction** | **Amino acids** |
| --- | --- | --- | --- |
| STAT3 | AA | Hydrogen | CYS251  ARG325  ASP334  SER514 |
|  |  | Hydrophobic | ALA250  PRO255  PRO256  ILE258  TRP510 |
|  |  | Van der Walls | GLN247  GLY253  GLY254  GLU324  GLN326  CYS328  PRO333  PRO336  GLU506  SER509  SER513 |
|  | AB | Hydrogen | GLN232  THR236  SER319  ASP237  GLU311  LEU312  ASN485 |
|  |  | Hydrophobic | ALA241  LYS244  PRO487 |
|  |  | Van der Walls | LEU240  ARG308  ASN315  LEU316  LYS318  GLU455  THR456  HIS457  LYS488 |

**Video S1** Molecular dynamic simulation between STAT3 and AA

**Video S2** Molecular dynamic simulation between STAT3 and AB
